# Identification of Highly Variable Supernumerary Chromosome Segments in an Asexual Pathogen

**DOI:** 10.1371/journal.pone.0158183

**Published:** 2016-06-24

**Authors:** Xiaoqiu Huang, Anindya Das, Binod B. Sahu, Subodh K. Srivastava, Leonor F. Leandro, Kerry O’Donnell, Madan K. Bhattacharyya

**Affiliations:** 1 Department of Computer Science, Iowa State University, Ames, Iowa, United States of America; 2 Plant Sciences Institute, Iowa State University, Ames, Iowa, United States of America; 3 Department of Agronomy, Iowa State University, Ames, Iowa, United States of America; 4 Crop, Soil and Environmental Sciences, University of Arkansas, Fayetteville, Arkansas, United States of America; 5 Department of Plant Pathology, Iowa State University, Ames, Iowa, United States of America; 6 National Center for Agricultural Utilization Research, US Department of Agriculture, Agricultural Research Service, Peoria, Illinois, United States of America; Ruhr-University Bochum, GERMANY

## Abstract

Supernumerary chromosome segments are known to harbor different transposons from their essential counterparts. The aim of this study was to investigate the role of transposons in the origin and evolution of supernumerary segments in the asexual fungal pathogen *Fusarium virguliforme*. We compared the genomes of 11 isolates comprising six *Fusarium* species that cause soybean sudden death syndrome (SDS) or bean root rot (BRR), and identified significant levels of genetic variation in A+T-rich repeat blocks of the essential chromosomes and in A+T-neutral regions of the supernumerary segments. The A+T-rich repeat blocks in the essential chromosomes were highly variable between *F. virguliforme* and non-*F. virguliforme* isolates, but were scarcely variable between *F. virguliforme* isolates. The A+T-neutral regions in the supernumerary segments, however, were highly variable between *F. virguliforme* isolates, with a statistically significant number (21 standard deviations above the mean) of single nucleotide polymorphisms (SNPs). And supernumerary sequence types and rearrangement patterns of some *F. virguliforme* isolates were present in an isolate of *F. cuneirostrum* but not in the other *F. virguliforme* isolates. The most variable and highly expressed region in the supernumerary segments contained an active DNA transposon that was a most conserved match between *F. virguliforme* and the unrelated fungus *Tolypocladium inflatum*. This transposon was absent from two of the *F. virguliforme* isolates. Furthermore, transposons in the supernumerary segments of some *F. virguliforme* isolates were present in non-*F. virguliforme* isolates, but were absent from the other *F. virguliforme* isolates. Two supernumerary P450 enzymes were 43% and 57% identical to their essential counterparts. This study has raised the possibility that transposons generate genetic variation in supernumerary chromosome segments by frequent horizontal transfer within and between closely related species.

## Introduction

Recent results from comparative genomics of closely related plant pathogens have revealed that genes in repeat-rich regions tend to evolve more rapidly than those in the rest of the genome [1–4]. A common type of rapid evolution was found in A+T-rich repeat blocks affected by repeat-induced point mutation (RIP) [5–7]. Similarly, the average non-synonymous substitution rate in the extra nonessential chromosomes (called supernumerary chromosomes) of the wheat pathogen *Mycosphaerella graminicola* was three times higher than in the essential chromosomes [8]. As another example, evidence of sexual reproduction has not been detected in many important plant pathogens [9, 10]. Asexual fungal pathogens are known to have variable electrophoretic karyotypes [11]. It was shown through pulsed-field gel eletrophoresis that supernumerary chromosomes were present only in some individuals of the asexual pathogen *Colletotrichum gloeosporioides* [12]. Furthermore, it was demonstrated under laboratory conditions that a 2-Mb supernumerary chromosome was transferred between two vegetatively incompatible isolates of *C. gloeosporioides* [13]. A whole-genome comparative study also pointed to horizontal transfer as the most likely origin for *F. oxysporum f.sp. lycopersici* supernumerary chromosomes [14]. In addition, the genome of the asexual pathogen *Verticillium dahliae* contained lineage-specific (LS) regions that were highly variable among isolates [15–17]. Previous studies in three species revealed that the supernumerary chromosomes contained different transposons from their essential counterparts [18]. These results motivated us to investigate the role of transposons in the origin and evolution of supernumerary chromosomes.

*Fusarium virguliforme* is an economically important fungal pathogen that causes sudden death syndrome (SDS) in soybean in North and South America [19]. The pathogen has reached all major soybean-growing areas in the USA since its first detection in 1971 [20]. Studies with various molecular markers detected an extremely low level of genetic variation within *F. virguliforme* isolates from North and South America [21, 22]. Moreover, mating experiments with 17 US isolates of *F. virguliforme* indicated that they all belonged to a single mating type [23]. However, different *F. virguliforme* isolates showed variation in aggressiveness on soybean plants, and seven karyotypic patterns were detected in 22 *F. virguliforme* isolates [24], suggesting the existence of supernumerary chromosome segments. A genome assembly of a *F. virguliforme* isolate was produced recently [25], and the mating type locus in *F. virguliforme* and its six close relatives were characterized. A polymerase chain reaction (PCR) assay based on both mating type sequences revealed that all 129 isolates of *F. virguliforme* in North and South America possessed the *MAT1-1* mating type [26]. These data suggest that the reproduction mode of *F. virguliforme* on soybean is asexual.

*F. virguliforme* is related to the sexual fungal pathogen *Nectria haematococca* MPVI, which possessed supernumerary chromosomes and repeat-rich regions [27]. These supernumerary chromosomes contained genes involved in degradation of plant antimicrobial compounds and in host-specific pathogenicity [18]. Sequences of the *N. haematococca* MPVI supernumerary chromosomes [28] can be used to determine if their homologs were present in *F. virguliforme*.

*F. virguliforme* is closely related to five morphologically distinct *Fusarium* species that cause SDS or bean root rot (BRR): *F. azukicola*, *F. brasiliense*, *F. cuneirostrum*, *F. phaseoli* and *F. tucumaniae* [29]. *F. tucumaniae* is the only known sexually reproducing fungus among these species [23]. In this study, we selected ten isolates of these closely related species—three (*F. virguliforme*), three (*F. tucumaniae*), and one (each of the other four species)—for next-generation genome sequencing (at a depth above 100) and analysis in comparison with the *F. virguliforme* Mont-1 genome [25] as a reference. The genome sequences of the ten isolates can be used to detect types and rates of inter- and intraspecific variation in the *F. virguliforme* genome. The four *F. virguliforme* isolates used in this study had a much lower SNP rate (0.00005, see Results for details) between them in the essential genome than most isolates used in previous studies. This SNP rate, for example, was at least 10 times lower than that between the two closest isolates used in [6]. This low background SNP rate along with a read coverage depth above 100 made it less problematic to find regions with elevated rates of real nucleotide differences between some of the isolates.

In this study, *F. virguliforme* isolate Mont-1 possessed genome segments that had significant unique matches to portions of *N. haematococca* MPVI supernumerary chromosomes. In addition, these genome segments were not present in some of the eleven isolates. Because of this isolate-specific property, these segments are referred to as supernumerary segments in this paper. On the other hand, essential genome segments are defined as those segments that had long portions present in all the eleven isolates. A significant portion of the essential genome of *F. virguliforme* isolate Mont-1 was A+T-rich repeat blocks. A major goal of the present study was to determine if supernumerary segments evolved much more rapidly than the essential genome (including A+T-rich repeat blocks) by investigating the extent of intraspecific variation in the *F. virguliforme* genome. We hypothesized that there were novel mechanisms to generate genetic variation rapidly in supernumerary segments. This high rate of variation in supernumerary regions may be revealed by looking at isolates with little variation in the essential genome. Another major goal of the present study was to determine whether interspecific transfers [18] might have been involved regarding the origin of *F. virguliforme* supernumerary regions by examining common supernumerary sequence types in some of the eleven isolates. The last major goal was to determine whether transposons played a role in the generation of genetic variation in supernumerary segments by examining essential and supernumerary transposons in all or some of the eleven isolates.

## Materials and Methods

### Isolates and sequence data

We selected ten isolates of six *Fusarium* species and produced Illumina paired-end reads of 102 bp for each of them. These whole-genome data are available at the NCBI Sequence Read Archive under accession PRJNA289542. Also available under this accession number is a transcriptomic data set of Illumina single reads of 83 bp from isolate *F. virguliforme* Mont-1. We previously produced a genome assembly (NCBI BioProject Accession: PRJNA63281) of isolate *F. virguliforme* Mont-1 [25], which was used as a reference genome assembly in this study. The origin, year of collection, and name abbreviation of each of these 11 isolates are presented in Table 1.

**Table 1:** Isolates used in this study.

Isolate^1^	Origin	Year	Abbreviation
*F. virguliforme* Mont-1	USA, Illinois	1991	*Fv* Mont-1
*F. virguliforme* Clinton-1B	USA, Iowa	1993	*Fv* Clinton-1B
*F. virguliforme* LL0009	USA, Iowa	2006	*Fv* LL0009
*F. virguliforme* NRRL 34551	Argentina, Buenos Aires	2002	*Fv* 34551
*F. cuneirostrum* NRRL 31157	USA, Michigan	1992	*Fc* 31157
*F. phaseoli* NRRL 31156	USA, Michigan	Unknown	*Fp* 31156
*F. brasiliense* NRRL 31757	Brazil, Distrito Federal	1992	*Fb* 31757
*F. tucumaniae* NRRL 31096	Argentina, Tucumán	2001	*Ft* 31096
*F. tucumaniae* NRRL 31781	Argentina, Tucumán	Unknown	*Ft* 31781
*F. tucumaniae* NRRL 34546	Argentina, Buenos Aires	2000	*Ft* 34546
*F. azukicola* NRRL 54364	Japan, Hokkaido	1997	*Fa* 54364

^1^ NRRL = Agricultural Research Service Culture Collection, National Center for Agricultural Utilization Research, USDA-ARS, Peoria, IL. No NRRL number is known for some isolates.

### Genomic DNA preparation, library construction and sequencing

The isolates were grown from single conidial spores. Each isolate was grown in 1/3 PDA for two weeks to get conidia. Harvested conidial spores were grown for 18 h in MSM medium to harvest mycelia for DNA preparation. The DNA from germinating conidial spores of the isolate was prepared by following a published protocol [30]. Then it was used for the construction of a paired-end library with an average insert size of 300 bp. For each isolate, over 59 million paired-end reads of 102 bp for a total of over 12 Gb were produced on Illumina HiSeq 2500 (Illumina, Inc. San Diego, CA, USA) at the DNA Facility, Iowa State University. No read trimming was performed; only reads with an end-to-end match (of a high percent identity) to the reference were selected in read mapping, and only reads having an end-to-end overlap (of a high percent identity) with another read were used in assembly.

### RNA extraction from germinating conidial spores and mycelia samples

The isolate *Fv* Mont-1 was maintained on Bilay media [(0.1% KH2PO4 (w/v), 0.1% KNO3 (w/v), 0.05% MgSO4 (w/v), 0.05% KCl (w/v), 0.02% starch (w/v), 0.02% glucose (w/v), 0.02% sucrose (w/v) and 2% agar(w/v)] plates and grown on 1/3 PDA [0.04% potato starch (w/v), 0.2% glucose (w/v), 2% agar (w/v)] to harvest conidial spores. The 1/3 PDA grown *Fv* Mont-1 became blue two weeks later when masses spores were produced. Harvested conidial spores were grown for 12 h in liquid modified Septoria medium (MSM) to obtain germinated spores [31]. The germinated spores were also continued to grow in the same medium for two weeks to obtain mycelia. Total RNAs were isolated from the germinating conidial spores and mycelia using TRIzol Reagent (Invitrogen, Carlsbad, CA, USA). The quality of RNA samples was determined by running RNAs on a denaturing agarose gel.

### cDNA library preparation for transcript sequencing

Total RNAs, 10 *μ*g each from germinating conidial spores and mycelia, were used to purify poly (A)+ RNAs using oligo (dT) attached to the magnetic beads (Promega, Madison, WI). RNA samples were reverse transcribed using a cDNA synthesis kit from Illumina (Illumina, Inc. San Diego, CA, USA), and cDNAs of an individual RNA sample were sequenced on Illumina Genome Analyzer II (Illumina, Inc. San Diego, CA, USA) at the DNA Facility, Iowa State University. Over 16 million single reads of 83 bp were generated using Solexa GA pipeline 1.6. No read trimming was performed; only reads with a high percent identity to the reference were selected in read mapping.

### Read mapping and SNP detection

A SNP between the reference isolate and another isolate (query) has two or more alleles termed REF and ALT. The REF allele refers to the allele in the reference and ALT alleles refer to alternate non-reference alleles. A SNP is of type 2 if both the REF allele and the ALT allele are present in the query isolate, and of type 1 if only the ALT allele is present in the query isolate. Because all the isolates in the vegetative state have haploid nuclei, the presence of type-2 SNPs in any of them indicates the presence of paralogous sequences in it. Thus, in some cases, type-2 SNPs were excluded in the calculation of the SNP rate between the reference and each query isolate.

The sets of Illumina paired-end reads for each query isolate were mapped onto the reference genome assembly with Bowtie2 [32]. The output from Bowtie2 in SAM format was redirected to Samtools [33] with the view command to produce output in BAM format, which was sorted with the sort command. The sorted output in BAM format was piled up on the reference with the mpileup command. The sorted BAM output files for all the isolates along with the reference genome assembly were uploaded into Integrative Genomics Viewer [34] for viewing SNPs and presence/absence polymorphisms in each isolate.

We used the following procedures to ensure that SNPs detected in this study were not due to errors in the sequence data or methods. First, the entire read must align to the reference with at least 95% percent identity. Second, a mapping quality cutoff of 40 (corresponding to a mapping error rate less than 1 in 10,000) was used. The use of this low read mapping error rate means that if a read was sufficiently identical to two or more regions of the reference, then the read was rejected by the mapping process. As a result, nearly identical regions of the reference were not covered by reads; we confirmed this mapping feature by finding such regions and checking on their read coverage depths. Note that reads with type-2 SNPs were still mapped to a unique region of the reference. Either there was only one copy of this element in the reference isolate, or highly identical copies (with over 99% identity) of the element were collapsed into one reference region during the assembly. For this reason, type-2 SNPs were not used in SNP rate calculations. Third, the SNP calls were filtered with a minimum quality value of 80 and a minimum read coverage depth of 10. Errors in SNP detection were unlikely to occur because of the high depths of coverage (over 100X) for the reference by reads from each isolate. In addition, we also used the FreeBayes-based SNP call component of SpeedSeq [35] to detect SNPs in the query isolates. Both methods produced highly similar results.

To ascertain that SNPs were not due to errors in the reference assembly by Srivastava et al. [25], this 454-read-based assembly for isolate *Fv* Mont-1 was evaluated by mapping Illumina reads from isolate *Fv* 34551; a read of 102 bp can be mapped to a region of the 454 assembly if they had at most 5 base differences. About 97.8% of the assembly was covered by reads at an average depth of 167. The genome-wide nucleotide difference rate for the 454 assembly was about 1 in 10,000 bp. This rate was an upper bound on the genome-wide error rate for the 454 assembly because the nucleotide differences comprised both errors and SNPs.

In addition, contigs mc28.2 and mc28.4 in the 454 assembly, which were highly variable between some *F. virguliforme* isolates, were evaluated to make sure that they had an error rate much lower than the variation rates. About 98% of contig mc28.2 was covered by mapped Illumina reads from isolate *Fv* 34551 to an average depth of 207 (S1 Fig). Only 5 nucleotide differences were found between contig mc28.2 and the mapped Illumina reads. A nucleotide difference was counted as an error if more Illumina reads differ from than agree with the contig base. Of the 5 differences, 2 were errors. Thus, the error rate was estimated to be 0.00004 (2 divided by the length of the contig read coverage). In contrast, the SNP rate for isolate *Fv* Clinton-1B in contig mc28.2 was 0.00614, 153 times higher than the error rate. Similarly, the estimated error rate for mc28.4 was 0.00003; the SNP rate for isolate *Fv* Clinton-1B was 0.00509, 169 times higher than the error rate (S2 Fig). Note that this estimated error rate was an overestimate since the SNPs may be counted as errors.

### Assembly of short reads

An assembly of paired-end reads for each isolate was performed with an Illumina version of PCAP (PCAP.Solexa) with the following data and options: a pair of mate files in fastq format; a minimum insert length of 100 bp and a maximum insert length of 700 bp; an average insert length of 400 bp with a standard deviation of 100. The minimum length of overlaps with no base mismatch match was set to 84 bp, and that of overlaps with up to three base mismatches was set to 90 bp. No overlap with more than three base mismatches was accepted. Each data set was of size up to 49 Gb, and each assembly could be produced in a day on a processor with 100 Gb of main memory.

About a dozen PCAP.Solexa contigs were selected for analysis in this study. These contigs were further checked by comparing them with the contigs produced by a popular short read assembler named SPAdes [36]. All but two of the PCAP.Solexa contigs had unique perfect matches to SPAdes contigs. The two contigs each had a difference with a SPAdes contig at one of the variants; these contigs were each completely covered at a high depth by some of the short reads when they were mapped onto the whole genome assembly (including the two contigs) with a minimum quality value of 40.

PCAP.Solexa still follows the overlap-layout-consensus strategy of PCAP [37]. It computes overlaps between Illumina reads using a more efficient alignment method. Then it builds contigs using read-level overlaps. All other Illumina read assemblers build contigs using overlaps between substrings of length k (called k-mers). Using k-mer overlaps trades the read-level consistency of contigs for efficiency and simplicity; a contig based on k-mer overlaps may have a region that is not entirely similar to any read.

### Assembly mapping

Each assembly of Illumina reads was mapped to the reference genome assembly by BWA-MEM [38] with the default options. The output from BWA-MEM in SAM format was redirected to Samtools [33] with the view command and -bS options to produce output in BAM format, which was sorted with the sort command. An output file of SNPs and indels in VCF format was produced in the same way as in the read mapping. The assembly mapping was useful in finding long indels between contigs in the reference assembly and query assembly, respectively. The coordinates of an indel between two contigs were found by computing an alignment of the contigs with GAP3 [39].

### Gene identification and functional annotation

Ab initio gene identification in a *Fusarium* genomic sequence was performed using Augustus [40] with training data from *F. graminearum*. A non-redundant protein sequence database at National Center for Biotechnology Information was searched using Blastx [41] with a genomic coding region as a query to find a set of protein database sequences that were most similar to the coding region. The gene structure from Augustus was refined by AAT [42] on the set of protein database sequences. Functional annotation of genes was performed using the HMMER web server [43].

### Estimation of duplications in a genome assembly

This was done by computing the duplication depth for each position in the genome assembly, where the duplication depth of a position was defined as the number of times a sufficiently long region containing the position was similar to another region in the genome assembly. The region length cutoff was set to 400 bp as duplications of lengths above this cutoff would be affected by RIP [44] in the lineage with past RIP activity. For example, if a contig had only two regions (with an overlap) of lengths above the cutoff that were each similar to somewhere else, then the duplication depth for each position in the contig was 2 if the position was inside the overlap, 1 if it was inside the non-overlapping part of either region, and 0 otherwise.

The duplication depths for the genome assembly were computed as follows. Initially, the duplication depth for each position was set to 0. A file of contig sequences for the genome assembly was compared with itself by modifying the DDS2 program [45] so that trivial matches of a contig sequence with itself was not reported and the symmetric matches for the same pair of similar regions of lengths above the cutoff were reported only once. Note that running the original DDS2 with two file arguments being the same genome file would result in two symmetric matches for a pair of similar regions between contigs A and B: one match formed with contig A from file one and contig B from file two, and the other match with contig B from file one and A from file two. The default match parameter values were used except for linear penalties for gaps. For each of the two regions in each reported match, the duplication depth for each position in the region was increased by 1. After the computation, the fraction of duplications in the genome assembly was estimated by dividing the number of positions with a positive duplication depth by the total number of positions.

### Phylogenetic analysis

A maximum-likelihood tree of the 11 SDS/BRR *Fusarium* isolates was inferred from genome-wide SNP data. The data were produced by mapping reads from each of 10 of the 11 isolates onto a genome assembly of the reference *Fv* Mont-1. A covered SNP position was a position of the reference that was sufficiently covered by reads from each isolate and had an alternative allele (a SNP) in the read coverage of this position from one of the 10 isolates. A total of 297,076 covered SNP positions were aligned in the 11 isolates. The multiple sequence alignment was analyzed to infer the tree with 200 bootstrap samples.

## Results

### High levels of variation in an A+T-rich and an A+T-neutral portion of the *F. virguliforme* genome

We mapped short reads from each of the ten isolates onto a 50-Mb genome assembly of isolate *Fv* Mont-1 [25] as a reference. The length of the reference covered by reads from the isolate and the distribution of type-1 SNPs (with no REF allele in the query isolate) between the reference and the isolate are given in Table 2. Table 2 reveals several patterns of genetic variation among the isolates. First, the four *F. virguliforme* isolates possessed a low genome-wide SNP rate of less than 1 in 10,000 bp, which is consistent with an asexual mode of reproduction. Isolate *Fv* 34551 collected in South America in 2002 was closer to *Fv* Mont-1 collected in the USA in 1991 than the other two *F. virguliforme* isolates collected in the USA. Second, the genome-wide SNP rate of about 1 in 200 bp between the reference and each non-*F. virguliforme* isolate was at least 80 ($<\frac{0.00409}{0.00005}$) times higher than that between the reference and each *F. virguliforme* isolate, indicating a significantly higher level of polymorphism and suggesting a much longer divergence time between *F. virguliforme* and the other species (see phylogenetic analysis below). Third, the genome-wide SNP rate of 1 in 200 bp between the reference and each non-*F. virguliforme* isolate was not high enough to explain why a large portion (∼10 Mb) of the reference genome was covered by reads from every *F. virguliforme* isolate, but not covered by reads from any non-*F. virguliforme* isolate.

**Table 2:** Length of coverage and distribution of SNPs when reads were mapped onto reference *Fv* Mont-1.

Isolate	Length of coverage (Mb)	Number of SNPs	Mean SNP rate/standard deviation^1^	Max SNP rate^2^
*Fv* 34551	49.9	3,269	0.00003/0.00003	0.00023 (6.7)
*Fv* Clinton-1B	49.6	6,315	0.00005/0.00028	0.00614 (21.8)
*Fv* LL0009	49.2	5,784	0.00005/0.00024	0.00620 (25.6)
*Fc* 31157	39.5	173,647	0.00442/0.00122	0.00803 (3.0)
*Fp* 31156	40.0	174,859	0.00441/0.00122	0.00806 (3.0)
*Fb* 31757	39.3	170,590	0.00432/0.00117	0.00883 (3.9)
*Ft* 31096	39.3	180,384	0.00459/0.00127	0.00934 (3.7)
*Ft* 31781	39.2	171,672	0.00439/0.00113	0.00817 (3.4)
*Ft* 34546	38.9	155,453	0.00409/0.00101	0.00723 (3.1)
*Fa* 54364	37.9	185,286	0.00501/0.00118	0.00943 (3.8)

^1^ The mapped reference was partitioned into at least 700 disjoint windows each with 35-kb sufficiently covered base positions. The mean and standard deviation were calculated for the SNP rates of these windows.

^2^ The number in the parentheses is the maximum SNP rate measured in units of standard deviation above the mean.

**Fig 1:**
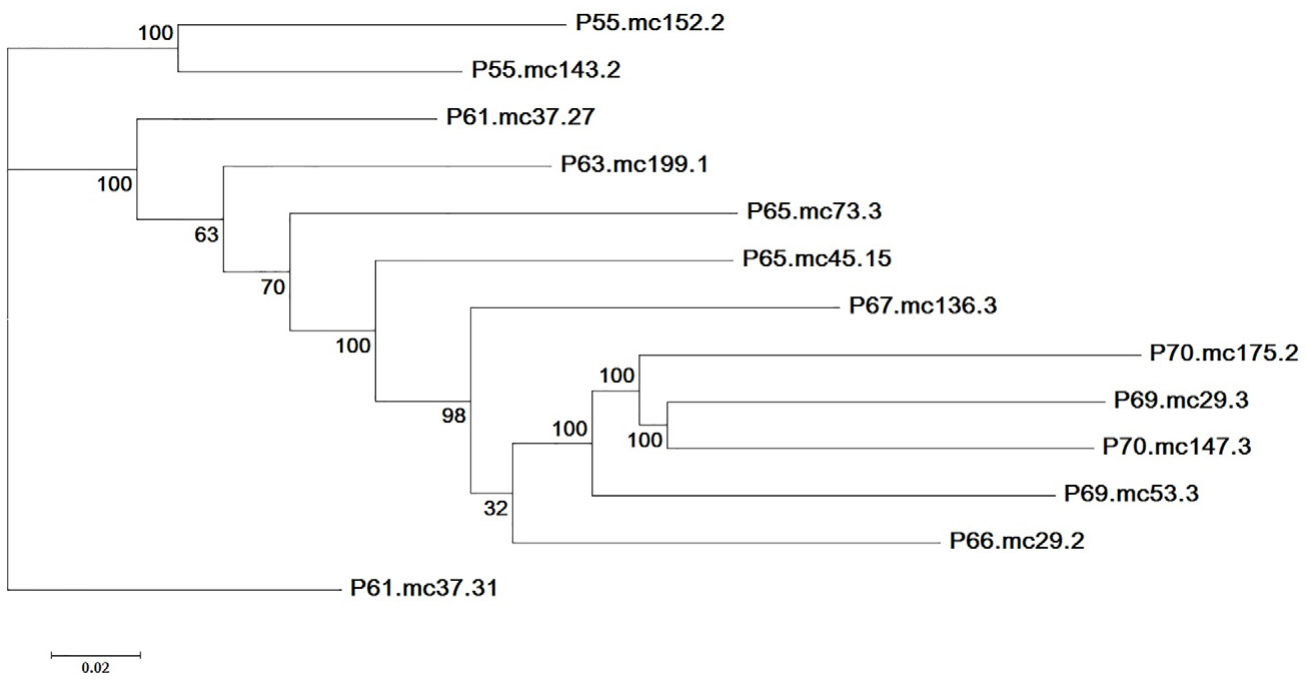
Maximum likelihood tree of 13 duplicated sequences (length 3,772 bp) in the *Fv* Mont-1 assembly. Each sequence was named based on its A+T content followed by its contig name. For example, sequence P61.mc37.31 indicates an A+T content of 61% and mc37.31 as its source contig. The four most recently duplicated sequences were P69.mc53.3, P70.mc175.2, P69.mc29.3 and P70.mc147.3, with the highest A+T content range of 69% to 70%. Support values from 100 bootstrap replicates are provided at internodes.

To shed light on the last observation, we selected all of the contigs that were at least 1 kb in the reference assembly and calculated the total number of contig bases covered by reads from *Fc* 31157 as well as that not covered by reads from this isolate. The size of the covered portion was 39.5 Mb; that of the uncovered portion was 10.9 Mb. The uncovered portion was A+T rich (68%), whereas the covered portion was A+T poor (45%). The content of duplicated sequences in the uncovered portion was 70%, with 48% containing sequences with copy numbers above 20. In sharp contrast, the content of duplicated sequences in the covered portion was 3.8%, with 0.56% containing 20-plus-copy sequences. These results indicate that *F. virguliforme* and *F. cuneirostrum* were more diverged in the uncovered portion rich in duplication and A+T content. For example, we observed C-T/G-A substitutions at a rate of 98% (871/887) in a 10-kb alignment (with 91% identity) of two adjacent contigs in the uncovered portion, where the integrity of both *Fv* Mont-1 contigs were verified based on their nearly complete and SNP-free coverage at a depth above 100 by reads from each of the other three *F. virguliforme* isolates. Each contig had a best match (with 90% identity) to the *Fc 31157* genome assembly and to the *Fp 31156* genome assembly, which were 99.95% identical over the match. Moreover, a maximum likelihood tree of 13 duplicated sequences in the *Fv* Mont-1 assembly illustrates that the more recently duplicated sequences had a higher A+T content (Fig 1).

The above observations indicate that the A+T-rich repeat blocks in the essential chromosomes were highly variable (at an estimated rate of 0.10) between the *F. virguliforme* and non-*F. virguliforme* isolates (with a genome-wide SNP rate of ∼0.005 (Table 2)), but were nearly invariable between the *F. virguliforme* isolates with a genome-wide SNP rate of ∼0.00005. Some of the A+T-rich repeat blocks in the essential chromosomes were about 45% identical at the amino acid level to reverse transcriptases with many in-frame stop codons.

Although the genome-wide SNP rate between the reference *F. virguliforme* isolate and each of the other three *F. virguliforme* isolates was at most 0.00005, we found high levels of variation among three of the four *F. virguliforme* isolates in a small portion (≤ 4%) of the genome; the maximum SNP rate between the reference *F. virguliforme* isolate and each of the two *F. virguliforme* isolates (*Fv* Clinton-1B and *Fv* LL0009) was at least 21 standard deviation units above the mean SNP rate. In addition, the maximum SNP rate for isolate *Fv* LL0009 was close to that for each of the non-*F. virguliforme* isolates, three of which belonged to the sexual species *F. tucumaniae*. This suggests that different evolutionary forces may have shaped their genomes.

The maximum SNP rates for both *Fv* Clinton-1B and *Fv* LL0009 with the reference assembly were observed in one of the two contigs (the second contig of 52,027 bp and with an A+T content of 49% and the fourth contig of 68,285 bp and with an A+T content of 49%) in scaffold 28 of the reference assembly. Scaffold 28 contained 12 contigs (with a total length of 217,558 bp and an overall A+T content of 49%) that were ordered and oriented using 454 read pairs with two insert sizes of 3 kb and 20 kb [25]. The two contigs, referred to as mc28.2 and mc28.4 (m for Mont-1 and c for contig), were separated by the third contig (referred to as mc28.3) of 36,918 bp. Scaffold 28 was linked by 14 read pairs (with an insert size of 20,000 bp) downstream to scaffold 66 with three contigs, the largest one of which was contig mc66.3 of 27,852 bp and with an A+T content of 49%. Many SNPs were also found in mc66.3 in each of the top six isolates of Table 2. Thus, the contig sequences in scaffold 66 were added to those in scaffold 28 to indicate that both scaffolds were from the same A+T-neutral supernumerary segment.

We also observed a significant number of type-2 SNPs in contigs mc28.2 and mc28.4 between the reference and each of the top six isolates in Table 2. The maximum type-2 SNP rate between the reference and each of the three *F. virguliforme* isolates was at least 0.00117. The high type-2 SNP rates indicated that two or more sequence types (paralogous sequences) were present in each *F. virguliforme* isolate. (Because of this, type-2 SNPs were excluded in Table 2.) In addition, high type-2 SNP rates in the A+T-neutral portion of the genome were found in the isolates of *F. cuneirostrum*, *F. phaseoli* and *F. brasiliense*, whereas low type-2 SNP rates in every region of the genome were observed in the isolates of *F. tucumaniae* and *F. azukicola*.

**Fig 2:**
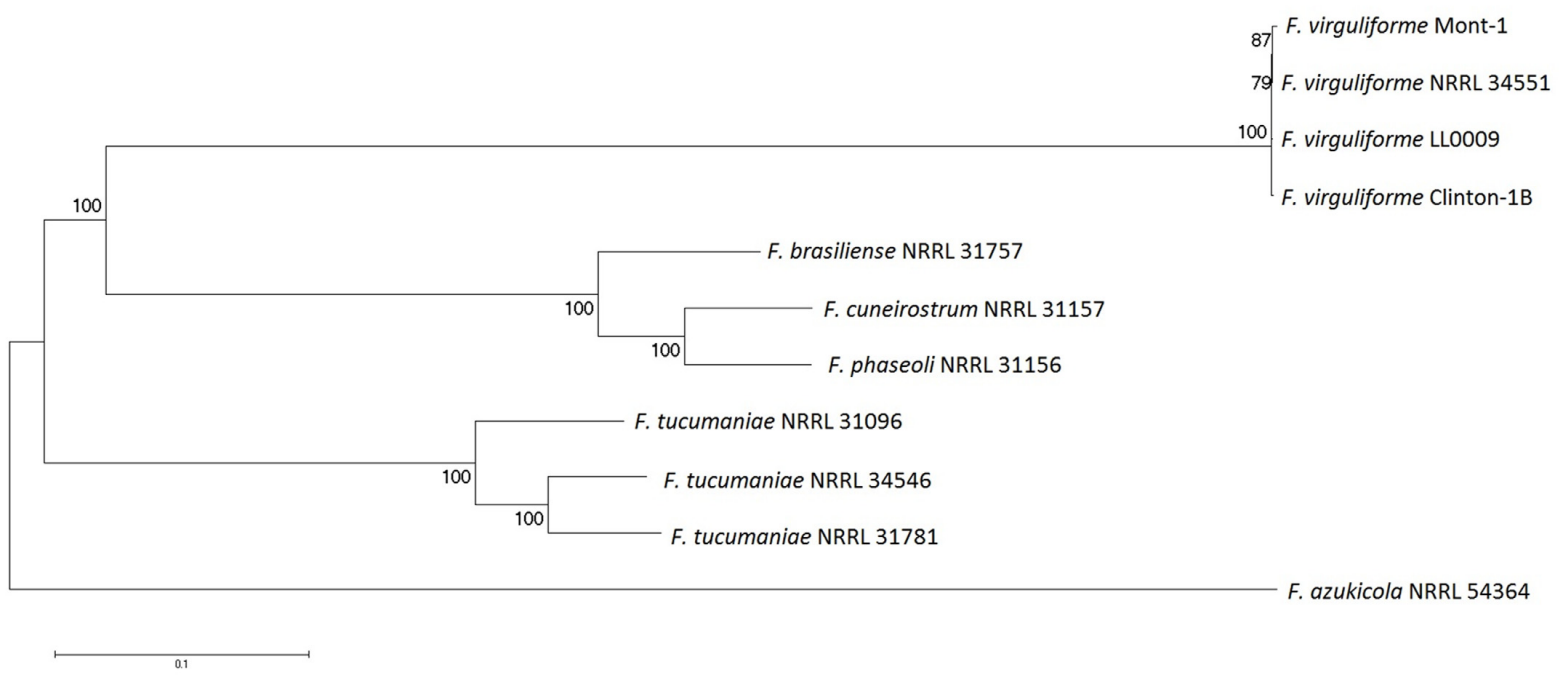
Maximum-likelihood midpoint rooted tree of 11 SDS/BRR *Fusarium* isolates, inferred from genome-wide SNP data with 200 bootstrap samples.

We inferred evolutionary relationships among the 11 isolates by constructing a phylogenetic tree (Fig 2) based on concatenation of 297,076 SNPs from the common reference assembly section sufficiently covered by reads from each isolate. The tree showed three clearly separate clusters: a first one formed by the four *F. virguliforme* isolates; a second one by *Fb* 31757, *Fc* 31157, and *Fp* 31156; a third one by the three *F. tucumaniae* isolates. The four *F. virguliforme* isolates formed a close cluster with extremely low levels of genome-wide variation among them. On the other hand, high levels of genome-wide variation were observed within the sexually reproducing species *F. tucumaniae*.

### The A+T-neutral portion was homologous to a known supernumerary chromosome

Scaffold 28 of the *Fv* Mont-1 genome assembly was compared (using DDS2 [45]) with the genome assembly of *Nectria haematococca* MPVI, the most closely related species whose genome sequence was determined previously [28]. Two unique significant matches (with at least 90% identity over 10,000 bp) were found in chromosome 14 of *N. haematococca* MPVI, a known supernumerary chromosome; one match was in mc28.4 and the other in mc28.10. The sequence of chromosome 14 was compared with the rest of the *Fv* Mont-1 assembly to find additional strong matches. No match meeting the above requirement was found; we found only one additional match (with 95% identity over 5,000 bp) in contig mc71.1. Like mc28.4, mc71.1 was rich in SNPs for some *F. virguliforme* isolates (see below). The unique significant matches between scaffold 28 of *Fv* Mont-1 and chromosome 14 of *N. haematococca* MPVI suggest that scaffold 28 was supernumerary. Thus, scaffold 28 is called a supernumerary segment.

Scaffold 28 was also highly variable among the *F. virguliforme* isolates, with several presence/absence polymorphisms. For example, contig mc28.3 was fully covered by reads from *Fv* 34551 with no SNPs, mostly covered by reads from *Fv* LL0009 with many SNPs, but barely covered by reads from *Fv* Clinton-1B. In addition, mc28.3 was highly variable among *Fc* 31157, *Fp* 31156, and *Fb* 31757. Similarly, contigs mc28.8 of 5 kb, mc28.11 of 8 kb, and mc28.12 of 8 kb were highly variable among the *F. virguliforme* isolates.

### The A+T-neutral portion contained sequence types shared by isolates of different species but not by isolates of the same species

Contigs mc28.2 (of 52 kb) and mc28.4 (of 68 kb) were compared with a genome assembly of each isolate to find corresponding contigs in the assembly with unique significant matches (with ≥ 94% identity over ≥ 5 kb), where the cutoffs for sequence similarity were selected based on the identity and length ranges of the unique significant matches between mc28.4 and the genome assembly of *Fv* 34551. Corresponding contigs were found in each of the top six isolates in Table 2. In addition, mc28.2 and mc28.4 were sufficiently covered by reads from each of these isolates. However, mc28.2 and mc28.4 were barely covered by reads from any of the bottom four isolates in Table 2 (see S1 and S2 Figs). In addition, little variation in mc28.2 was detected between the reference isolate and *Fv* 34551. For *Fv* 34551, the major differences in read coverage depth and type-2 SNP number between mc28.2 and mc28.4 indicate the presence of a long segment and a short segment in *Fv* 34551 that were highly polymorphic over mc28.4.

**Fig 3:**
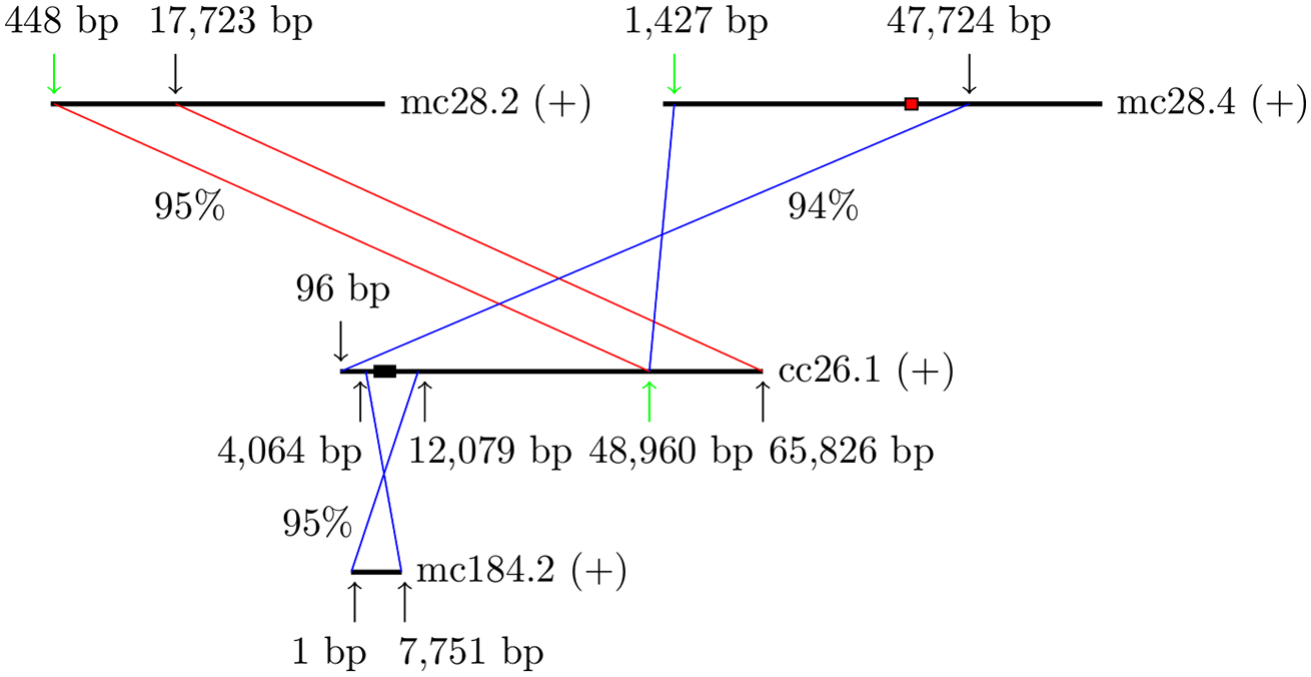
Chromosomal rearrangement between *Fv* Mont-1 and *Fv* Clinton-1B. Each horizontal line represents a contig with its name and orientation (+ denotes forward) given on the right. A unique significant match between contig regions in opposite orientations is indicated by a pair of cross lines; one in the same orientation by a pair of parallel lines. In each case, the percent identity of the match is shown next to the lines. The beginning and end of each contig region in the match are marked with vertical arrows along with their positions in bp. A red box in contig mc28.4 and a black box in contig cc26.1 represent different islands surrounded by the match; the black box is part of the match with contig mc184.2.

By contrast, we detected significant variation in mc28.2 and mc28.4 between the reference isolate and *Fv* Clinton-1B by finding unique significant matches in a comparison of these contigs with the *Fv* Clinton-1B genome assembly. Some of the matches suggest a chromosomal rearrangement between the reference isolate and *Fv* Clinton-1B, and the presence of two genomic segments in the reference isolate that were 95% identical over some of their lengths but were quite different over the rest (Fig 3). The sequence integrity of cc26.1 over the breakpoint (marked by a green arrow in Fig 3) was confirmed by a match of 96% identity between a region of cc26.1 from 28,492 to 52,548 bp and a region of a contig of 27,382 bp from a genome assembly of *Fv* LL0009; the percent identity of the match around the breakpoint was nearly 99%. In addition, by mapping short reads from each isolate onto the *Fv* Clinton-1B genome assembly, we found that cc26.1 was covered at a depth above 200 over the breakpoint by reads from the five isolates: *Fv* Clinton-1B (at a depth of 414), *Fv* LL0009 (319), *Fc* 31157 (722), *Fp* 31156 (494), and *Fb* 31757 (231). However, cc26.1 was not covered at the breakpoint by any reads from *Fv* 34551, although cc26.1 was deeply covered before and after the breakpoint by these reads. Therefore, the rearrangement type in cc26.1 of *Fv* Clinton-1B was not present in *Fv* 34551; the rearrangement type in mc28.2 and mc28.4 of *Fv* Mont-1 was present only in *Fv* 34551 based on the deep read coverage of mc28.2 around the breakpoint (at a depth of 240) and of mc28.4 around the breakpoint (231). Furthermore, a type-2 SNP G/A (G, REF allele; A, ALT allele) was found near the breakpoint in cc26.1 in *Fv* Clinton-1B (G at a coverage depth of 253; A at 153), *Fc* 31157 (567/179), and *Fp* 31156 (278/224), a sign that two polymorphic segments were present in each of these three isolates.

**Fig 4:**
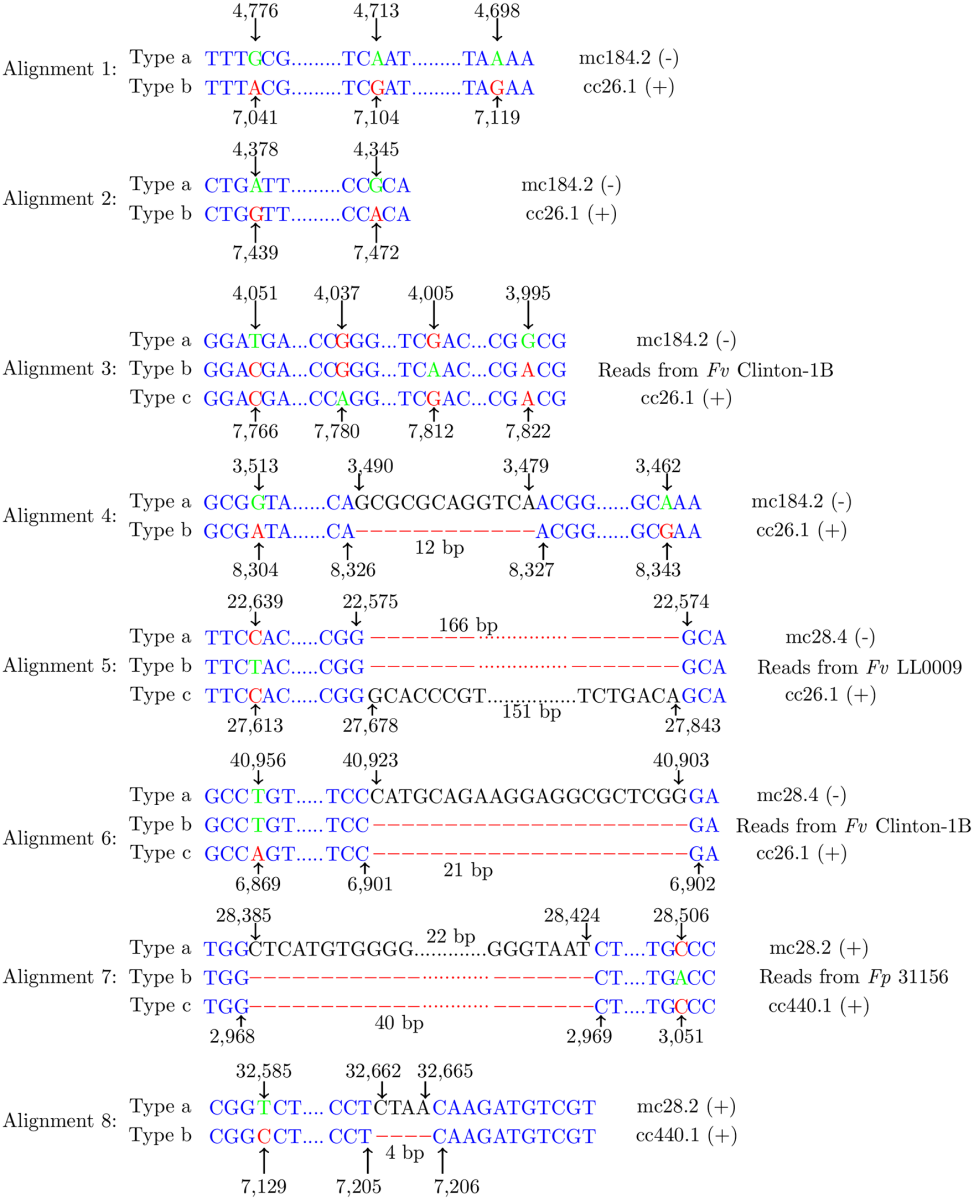
Eight sequence alignments with SNPs and small indels (4 to 166 bp). Each alignment is composed of two or three sequence types (denoted by Types a, b and c): a reference contig, a contig in the *Fv* Clinton-1B assembly, and sometimes short reads from one of the ten isolates, which were mapped to one of the two contigs. The name of each contig along with its orientation (+ denotes forward and—denotes reverse), or the name of the isolate if present, is shown to the right of its sequence type. Every allele in the contig is marked with an arrow and a number in bp showing its position. Notation: mc184.2, *Fv* Mont-1 contig 184.2; cc26.1, *Fv* Clinton-1B contig 26.1.

A total of eight contig sequence alignments showing SNPs and small indels between the reference isolate and *Fv* Clinton-1B are shown in Fig 4. Each alignment contained two or more instances of polymorphism, all of which were close enough to be linked by 102-bp reads. We checked for the presence/absence of these polymorphic sequences in each of the top six isolates in Table 2. This was done by mapping short reads from each of the six isolates onto the genome assembly of the reference isolate and again onto that of *Fv* Clinton-1B. We found additional types of polymorphic sequences by examining the read coverage of each contig sequence. Thus, some alignments in Fig 4 contained three polymorphic sequences. For each isolate and for each sequence in each alignment, Table 3 shows the number of reads from the isolate that matched and linked all alleles in the sequence. Note that for some isolates and alignments, the read counts from the isolate for each sequence in the alignment were quite different (e.g. a range of 33-78 reads per sequence for isolate *Fv* Clinton-1B and Alignment A3). The most likely explanation for the large difference in read count between the three types in isolate *Fv* Clinton-1B is that at least 4 copies of the supernumerary segment were present in this isolate, with type A3.Tb present in more copies than types A3.Ta and A3.Tc. In light of this observation, a segment with two or more highly polymorphic copies in an isolate is also called supernumerary. Thus, the detection of a significant number of type-2 SNPs in an isolate indicates the presence of a supernumerary segment.

**Table 3:** The number of reads from the isolate that link all alleles in the sequence.

	Number of reads from the isolate that cover the sequence^1^					
Sequence^2^	*Fv* Clinton-1B	*Fv* LL0009	*Fv* 34551	*Fc* 31157	*Fp* 31156	*Fb* 31757
A1.Ta	16	16	16	0	0	0
A1.Tb	32	18	0	48	46	8
A2.Ta	88	149	147	0	0	9
A2.Tb	85	114	0	115	0	0
A3.Ta	52	41	61	0	0	0
A3.Tb	78	52	0	152	0	0
A3.Tc	33	0	0	34	46	0
A4.Ta	162	134	77	121	0	97
A4.Tb	54	0	0	127	242	0
A5.Ta	39	27	57	0	0	18
A5.Tb	0	8	0	39	0	0
A5.Tc	85	0	0	69	65	0
A6.Ta	0	0	46	0	0	0
A6.Tb	72	0	0	0	209	0
A6.Tc	116	121	35	554	244	74
A7.Ta	0	0	98	0	0	0
A7.Tb	0	31	0	0	54	0
A7.Tc	35	0	0	42	50	39
A8.Ta	0	35	34	40	42	0
A8.Tb	70	0	0	51	59	42

^1^ A read covered a sequence in a set of polymorphic sequences if the read and the sequence had the same allele at every occurrence of polymorphism.

^2^ Each sequence is denoted by its alignment number and type letter (Fig 4): e.g., Types a and b in Alignment 1 are denoted by A1.Ta and A1.Tb, respectively.

Table 3 shows that every isolate except *Fb* 31757 contained two or more polymorphic sequence types, i.e., two or more copies of an element. A separate analysis of *Fb* 31757 revealed that it contained two alleles at each SNP position in its deep read coverage (≥ 500) of two large regions of mc28.4, a sign that the isolate contained two copies of an element. These observations suggest the possibility that copies of the element in scaffold 28 were transferred horizontally.

After discovering the short common sequence types in cc26.1 and cc440.1 between *Fv* Clinton-1B and *Fc* 31157, we checked to see if the two isolates were closer in the whole contigs than the other isolates. Contig cc26.1 was completely covered at a high depth by reads from *Fc* 31157, but only partially at a high depth by reads from each of the other four isolates. Thus, *Fv* Clinton-1B was most similar to *Fc* 31157 in this contig, which is another species, and less similar to *Fv* LL0009 and *Fv* 34551 of its own species. We also made a similar observation regarding contig cc440.1. These observations also suggest the possibility that the segment (i.e., a chromosome or part of it) in contigs cc26.1 and cc440.1 of *Fv* Clinton-1B was horizontally acquired from another species. The presence of two or more DNA segments homologous to scaffold 28 and with numerous small and large variations in each of the top six SDS/BRR isolates (in Table 2) suggests that the rate of transposition was frequent in this clade of closely related species.

### Additional supernumerary segments

We discovered another reference contig (mc74.1 of 75 kb) in which a high SNP rate between the reference isolate and *Fv* LL0009 was observed; it was 4.8 standard deviation units above the mean SNP rate. Isolate *Fv* 34551 was most similar to the reference isolate in contig mc74.1, as indicated by a low SNP rate between them. Contig mc74.1 was the first of a three-contig scaffold of 80 kb. We found a total of 119 type-2 SNPs in the *Fv* LL0009 read coverage of mc74.1, suggesting that the isolate contained two or more polymorphic copies of the segment in mc74.1. Contig mc74.1 (over its separate regions) had unique significant matches (with 99% identity over 10 kb) to two contigs (lc25.1 of 33 kb, and lc220.1 of 18 kb) in the *Fv* LL0009 genome assembly. Contig lc220.1 was a nearly perfect match over its whole length (except its short ends) to a region of mc74.1. However, contig lc25.1 was only a local match to mc74.1; a region of lc25.1 from positions 9,409 to 27,568 bp was 99% identical to a region of mc74.1 from positions 41,232 to 23,076 bp (in reverse orientation). Moreover, only this region of lc25.1 was covered in high depth by reads from *Fv* Mont-1, *Fv* 34551, *Fc* 31157, and *Fp* 31156.

On the other hand, contig lc25.1 from positions 4,845 to 12,262 bp was 99% identical to contig cc714.1 (from positions 7,419 to 1 bp) of *Fv* Clinton-1B; contig lc25.1 from positions 4,830 to 13,682 bp was 99% identical to contig bc299.1 from positions 8,853 to 1 bp of *Fb* 31757. The two strong matches confirmed the integrity of the region of contig lc25.1 from positions 4,830 to 9,408 bp. In addition, a region of lc25.1 from positions 606 to 4,153 bp was 99% identical to contig bc2776.1 (from positions 2 to 3,537 bp) of *Fb* 31757. This region of lc25.1 was not covered by any read from the other isolates. Contig cc714.1 was another contig in which not all of the six SDS/BRR isolates were the same in their read coverage of this contig.

We screened the reference assembly for additional contigs with a high SNP rate or contigs in which some of the isolates were different in their read coverage of these contigs. A total of 18 scaffolds with such contigs were found (Table 4). These scaffolds were candidate supernumerary segments.

**Table 4:** Scaffolds with supernumerary segments.

Scaffold	Length (kb)	Contig with type-2 SNPs or coverage variation (CV)^1^
26	379	mc26.1 (CV: *Fv* LL0009, *Fv* 34551)
28	218	mc28.2 (SNPs: *Fv* Clinton-1B)
33	330	mc33.8 (CV: *Fv* Clinton-1B, *Fv* LL0009)
41	207	mc41.8 (SNPs: *Fv* LL0009)
46	158	mc46.2 (CV: *Fv* LL0009, *Fv* 34551)
50	140	mc50.4 (SNPs: *Fv* Clinton-1B)
54	120	mc54.3 (CV: *Fv* Clinton-1B, *Fv* LL0009)
58	96	mc58.2 (CV: *Fv* Clinton-1B, *Fv* LL0009)
71	85	mc71.2 (SNPs: *Fv* Clinton-1B)
74	80	mc74.1 (SNPs: *Fv* LL0009)
79	73	mc79.6 (SNPs: *Fv* LL0009)
80	69	mc80.1 (CV: *Fv* LL0009, *Fv* 34551)
88	51	mc88.6 (SNPs: *Fp* 31156)
90	61	mc90.7 (CV: *Fv* 34551, *Fc* 31157)
91	44	mc91.1 (SNPs: *Fp* 31156)
98	45	mc98.3 (CV: *Fv* Clinton-1B, *Fv* LL0009)
99	34	mc99.2 (CV: *Fv* 34551, *Fv* Clinton-1B)
100	37	mc100.2 (CV: *Fv* Clinton-1B, *Fv* LL0009)
117	24	mc117.2 (CV: *Ft* 31096, *Ft* 31781)
158	12	mc158.2 (CV: *Fc* 31157, *Fp* 31156)

^1^ Shown in the parentheses are the names of two isolates in which read coverage variation was detected in the contig or the name of an isolate in which type-2 SNPs were detected in the contig.

### Genes in supernumerary segments

**Fig 5:**
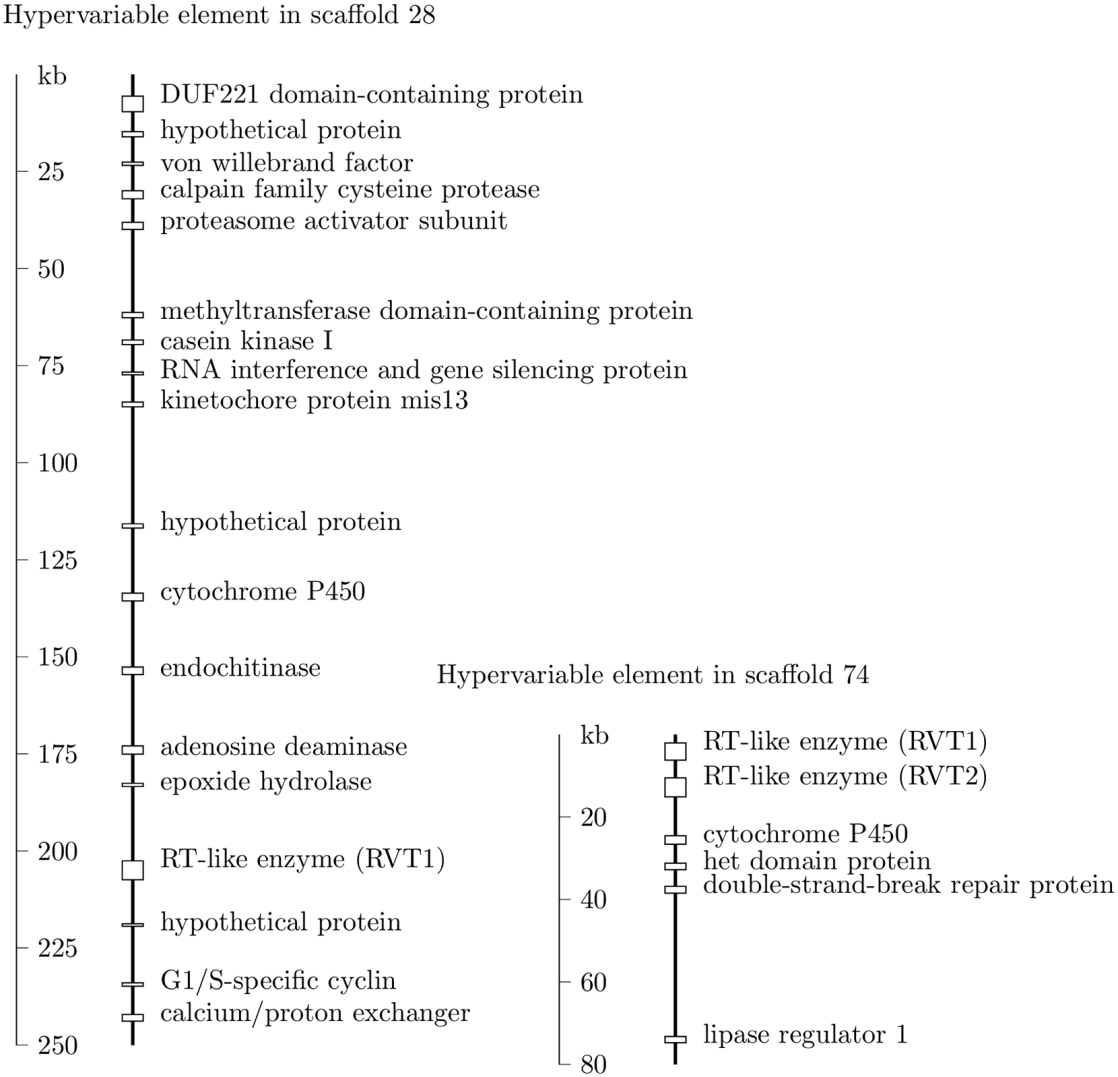
Proteins encoded by two supernumerary segments. The related proteins between the segments were P450 enzymes and reverse transcriptase-like (RT-like) enzymes. The larger segment contained a gene encoding a G1/S-specific cyclin protein.

We annotated genes in two supernumerary segments by combining ab initio gene structure prediction with protein database matching. A list of proteins along with their functions in each segment are shown in Fig 5. Some proteins were involved in drug metabolism, for example, cytochrome P450 and epoxide hydrolase. Others were related to cell cycle (e.g., cyclin), cell calcium control (e.g., calcium exchanger), cell wall (e.g. endochitinase), DNA replication (e.g., reverse transcriptase-related enzyme) and repair (e.g., double-strand-break-repair protein).

We examined variation in some of the genes among the isolates. Contig mc74.1 harbors a gene encoding a cytochrome P450 (CYP) enzyme of 643 residues. This enzyme, a member of family CYP53 (e-value = 1.0e-152), is capable of detoxifying plant defensive compounds, including benzoic acid derivatives [46]. The gene was present in the top seven isolates (in Table 2) including *Ft* 31096, but not in the other three isolates including *Ft* 31781 and *Ft* 34546 (S3 Fig). No SNPs were found in this gene in each of *Fv* Clinton-1B, *Fv* 34551, *Fc* 31157, *Fp* 31156, whose reads covered the reference locus at depths between 70 and 380; 2 SNPs were found in *Fb* 31757. In contrast, we found 11 type-2 SNPs in *Fv* LL0009. Of the 11 SNPs, 8 were nonsynonymous, 1 synonymous, 1 in an intron, and 1 in a 5′ untranslated region (UTR). In addition, in *Ft* 31096, 12 SNPs were found, of which 8 were nonsynonymous.

The supernumerary *CYP53* gene was 43% identical at the amino acid level to another region (contig mc2.51) in the essential (core) genome, where the two genes shared the same 4-exon gene structure with two short exons followed by two long exons. The core *CYP53* gene was present in all of the isolates with no SNPs among the *F. virguliforme* isolates and a total of 12 SNPs between the *F. virguliforme* isolates and the non-*F. virguliforme* isolates. Of the 12 SNPs, 3 were present in all the non-*F. virguliforme* isolates, 2 were in all the *F. tucumaniae* isolates, 3 were in *Fc* 31157 and *Fb* 31757, 3 were only in *Fa* 54364, and 1 was only in *Fp* 31156. The core CYP53 enzyme was 90% identical to a CYP53 enzyme of 635 residues from *N. haematococca* MPVI; which was also the best match (at 43% identity) for the supernumerary CYP53 enzyme when searched against all of the *N. haematococca* MPVI proteins.

Downstream of the supernumerary *CYP53* gene in contig mc74.1 was a 2-exon gene encoding a 514-residue protein with a heterokaryon incompatibility (HET) domain (e-value = 4.3e-17). Variation in the supernumerary *HET* gene among the isolates was similar to that of the supernumerary *CYP53* gene. The *HET* gene was present in the top seven isolates (in Table 2) with no SNPs in each of *Fv* Clinton-1B, *Fv* 34551, *Fp* 31156, and *Fb* 31757, and with 1 SNP in *Fc* 31157. We found 8 type-2 SNPs in *Fv* LL0009 with 6 of them being nonsynonymous, and another 8 type-1 SNPs in *Ft* 31096 with 6 of them being nonsynonymous. The supernumerary *HET* gene was a unique strong match (72% at the amino acid level over the full length) to a 2-exon gene in the essential genome (contig mc29.14), which was present in all of the isolates with no SNPs in each *F. virguliforme* isolate but with several SNPs in each non-*F. virguliforme* isolate.

We found another P450 enzyme (of 517 residues) in the supernumerary genome. The enzyme was encoded by a 4-exon gene in contig mc28.4, with two short exons followed by two long exons. The supernumerary P450 enzyme was 68% (342/497) identical to a P450 enzyme of *F. oxysporum* (FOXG_17536) in subfamily CYP567F; family CYP567 was merged with family CYP60 [47], a group of P450 enzymes involved in biosynthesis of secondary metabolites such as mycotoxins [48]. This *CYP567* gene was present with 11 to 21 SNPs in each of the top six isolates in Table 2. For example, we found 21 SNPs in *Fv* LL0009 with 6 in introns and 15 in coding exons, of which, 7 were nonsynonymous and 8 synonymous. The six isolates contained 5 common SNPs, which were all in coding exons with 4 of them being nonsynonymous. In each of the top five isolates in Table 2, most of the SNPs were type 2, indicating that at least two highly similar copies of the *CYP567* gene were present in the isolate. As an example, in isolate *Fc* 31157, a type-2 SNP C/T (357/904) was found at position 45,570 bp in mc28.4. The frequency for the allele T was twice that for the allele C, suggesting that at least three copies of the gene were present in this isolate, with one copy having the C allele and the other two having the T allele. The supernumerary CYP567 enzyme was a unique strong match with 57% identity (298/517) to a P450 enzyme in the essential genome (contig mc11.8).

We found a single-exon gene coding for a 248-residue G1/S-specific cyclin. This gene was present in each of the top six isolates (in Table 2) with at least two copies in four of them: *Fv* Clinton-1B, *Fv* 34551, *Fc* 31157 and *Fp* 31156, based on the complete coverage of the reference gene sequence by reads from each isolate and the presence of at least 9 type-2 SNPs. For example, we observed a SNP C/T (at codon position 2 for residue 211) in each isolate’s read coverage of the reference gene sequence with the following read counts for each allele: 141/106 (*Fv* Clinton-1B), 0/132 (*Fv* LL0009), 384/147 (*Fv* 34551), 471/215 (*Fc* 31157), 417/239 (*Fp* 31156), and 0/172 (*Fb* 31757). Note that the count for the reference allele was about twice that for the alternate allele in three isolates, suggesting that there were three copies of this gene: two copies of the reference type and one copy of the alternate type. We found 9 type-2 SNPs in the *Fv* Clinton-1B read coverage of the reference gene sequence; most of the SNPs were present in the other isolates. Of the 9 substitutions, 6 were nonsynonymous and 3 were synonymous.

### Transposons in supernumerary segments

The supernumerary segment in scaffold 74 carried both *RVT1* and *RVT2* genes (Fig 5), which were conserved among the top six SDS/BRR isolates (in Table 2) based on the read coverage of the reference segment. The *RVT1* gene contained 4 predicted introns; the *RVT2* gene had one. The *RVT1* gene was predicted to encode a protein of 1,619 residues with an endonuclease/exonuclease (e-value = 3.2e-18) domain, a reverse transcriptase (3.1e-27), and an RNase H (8.9e-18). The endonuclease/exonuclease domain was in exon 4 encoding 430 residues, and the other two domains were mostly in exon 5 encoding 692 residues, with the two exons separated by an intron of 58 bp. The *RVT2* gene was predicted to encode a protein of 957 residues with an integrase core domain (4.6e-18) and a reverse transcriptase domain (2.9e-88) but without an endonuclease/exonuclease or RNase domain. The two domains were in exon 2 encoding 710 residues. Scaffold 74 had a G+C content of 52%. We searched the rest of the reference genome for strong matches to either RVT protein and found 7 additional *RVT1* regions and 3 additional *RVT2* ones.

For each region, we checked whether its scaffold was variable among the isolates, and if so, we checked whether the presence (or absence) of long *RVT* ORFs in the region was correlated with the presence (or absence) of type-1/2 SNPs in the read coverage of this region by some isolates. The results are shown in Table 5. Of the 7 scaffolds with an *RVT1* match (top 7 rows in Table 5), 5 (scaffolds 71, 28, 54, 88, 117) were supernumerary, and 2 (scaffolds 51 and 15) were parts of the essential genome. The 3 scaffolds with an *RVT2* match (bottom 3 rows in Table 5) were all supernumerary. Of the 8 supernumerary segments, 6 (scaffolds 71, 28, 54, 88, 41, 50) contained long *RVT* ORFs with a significant SNP rate, and 2 (scaffolds 117 and 57) contained shorter *RVT* ORFs with a lower SNP rate.

**Table 5:** SNP rates in 10 scaffolds with an RVT match and numbers of in-frame stop codons in the matches.

Scaffold	G+C content	Match length^1^	Match percent identity	Number of stop codons	Max SNP rate^2^
71	51%	1371	76%	0	0.00060
28	50%	1321	72%	0	0.00290
54	51%	1612	47%	0	0.00300
88	52%	1619	43%	3	0.00049
117	42%	1010	62%	9	0.00025
51	45%	668	71%	37	0.00006
15	52%	670	78%	0	0.00003
41	50%	957	91%	1	0.00180
50	50%	957	50%	1	0.00091
57	44%	845	43%	21	0.00009

^1^ The top 7 scaffolds contained a match to the RVT1 protein; the remaining 3 had a match to the RVT2 protein. The length of each match was in amino acids.

^2^ For each reference scaffold, the maximum of the type-1 and type-2 SNP rates for isolates *Fv* Clinton-1B, *Fv* LL0009 and *Fv* 34551 in the scaffold was calculated.

In addition, we compared the genome assemblies of isolates *Fv* Mont-1 and *Fv* Clinton-1B with the Repbase database [49] (release RepBase21.02) and found highly significant matches (e-values as low as 0): 18 matches in the *Fv* Mont-1 assembly and 36 matches in the *Fv* Clinton-1B assembly. All but one of these 54 matches were in supernumerary regions; the only match (of 642 bp and with an A+T content of 53%) not in the supernumerary regions was 91% identical (with two in-frame stop codons) to a 236-residue region of a 626-residue transposase (GenBank accession: AAC16005) from *F. oxysporum f. sp. lycopersici*. Nearly all of the matches in each assembly were with DNA transposons, and nearly each match had an A+T content between 46% and 50%. Below are reports of the presence/absence polymorphisms and SNP rates in these transposons among some of the isolates.

We checked if these transposons were present in each isolate by calculating their coverage breadths by reads from the isolate. We observed presence/absence polymorphisms in 29 of the 54 transposons among the three *F. virguliforme* isolates *Fv* Clinton-1B, *Fv* LL0009 and *Fv* 34551. Of the 29 transposons, 8 were present in *Fv* Clinton-1B (with at least 95% of the transposon covered by reads from *Fv* Clinton-1B), but not in any of the five isolates *Fv* LL0009, *Fv* 34551, *Fc* 31157, *Fp* 31156 and *Fb* 31757 (with 0% of the transposon covered by reads from the isolate). The percent coverage of each of the remaining 21 transposons by reads from each of the six isolates is shown in Table 6. More than half of the transposons in Table 6 had high coverage breadths by reads from some *F. virguliforme* isolates and some non-*F. virguliforme* isolates, but a low coverage breadth by reads from at least one *F. virguliforme* isolate, which raised the possibility that the transposons were transferred horizontally.

**Table 6:** Breadth of coverage of the transposon at a minimum depth of 25 reads^1^.

	Percent coverage (%) of the transposon by reads from each isolate					
Transposon^2^	*Fv* Clinton-1B	*Fv* LL0009	*Fv* 34551	*Fc* 31157	*Fp* 31156	*Fb* 31757
R_mc28.3	0	5	100	20	20	19
M_mc41.12	88	0	100	95	97	0
H_mc54.4	15	100	100	100	13	0
H_mc58.2	0	100	100	54	0	0
T_mc98.3	0	100	100	4	4	0
N1_cc61.1	100	0	0	0	100	0
N1_cc116.1	98	0	0	89	99	95
H_cc168.1	100	94	0	0	0	0
M_cc367.1	100	69	80	87	0	0
F_cc390.1	100	98	63	57	75	58
M_cc435.1	100	0	0	100	100	0
T_cc487.1	100	60	8	100	100	0
N1_cc513.1	99	17	0	26	84	16
N1_cc541.1	98	0	0	99	99	57
P_cc749.1	99	19	0	98	99	99
H_cc781.1	100	100	0	100	98	0
N2_cc837.1	99	69	2	0	0	0
M_cc1408.1	100	68	66	0	0	0
P_cc3473.1	98	55	23	94	67	98
M_cc4313.1	100	100	0	100	100	98
M_cc7644.1	100	0	92	100	100	0

^1^ The minimum depth was set to a value such that the read depths at over 95% of all covered reference genome positions were above the value (25 reads).

^2^ Each transposon is denoted by its family name initial, an underscore and the name of the reference contig containing it. The initials for transposon family names: F, FOLYT1; H, Hop; M, MarCry-1_FO; N1, NHT1; N2, NHT2_I; P, PiggyBac-1_Nha; R, Restless; T, TFO1_FO.

We examined the DNA transposon Restless in the contig mc28.3, which was downstream of the contig mc28.2 and upstream of the contig mc28.4; as noted above, both mc28.2 and mc28.4 showed significantly higher SNP rates between some of the *F. virguliforme* isolates. The DNA transposon in mc28.3 was 91% identical over a region of 1,227 bp to the Restless transposon in Repbase, which came from the fungus *Tolypocladium inflatum* with a genome size of about 30 Mb. This match was one of the top three matches between the *F. virguliforme* and *Tolypocladium inflatum* genomes; the average percent identity of the 306 most significant matches of lengths over 1,000 bp between the two genomes was 79%. The Restless transposon in mc28.3 was highly expressed; its expression level ranked in the top 2% of all the genes. The G+C content of this transposon was 51%.

We also observed significantly higher SNP rates in or around 20 of the 54 transposons between some of the four *F. virguliforme* isolates *Fv* Mont-1, *Fv* Clinton-1B, *Fv* LL0009 and *Fv* 34551. The two most significant examples were a SNP rate of 0.004 in contig mc28.3 with the Restless transposon between *Fv* Mont-1 and *Fv* LL0009, and a SNP rate of 0.006 in contig cc32.1 with an NHT1 transposon between *Fv* Clinton-1B and *Fv* LL0009.

### Supernumerary segment in scaffold 28 was most highly expressed

We examined the expression levels of supernumerary segments because they contained *RVT* genes, which polymerize DNA via an RNA intermediate. The expression level of each contig in the *Fv* Mont-1 reference assembly was estimated by totaling the coverage depths of each contig position by short transcriptomic reads and dividing the sum by the contig length. The 11 most highly expressed contigs of ≥ 1 kb were mc66.3 (at an average depth of 144), mc4.6 (137), mc28.4 (130), mc28.8 (95), mc420.2 (88), mc79.8 (83), mc28.7 (81), mc54.5 (80), mc28.10 (74), mc28.12 (72), and mc28.2 (70). All the contigs except mc4.6 were supernumerary. Moreover, 6 of them came from scaffold 28, and 3 of the rest (mc66.3, mc420.2, mc79.8) were linked to scaffold 28 by a significant number of read pairs. In addition, we estimated the expression level of each gene in the same reference assembly. Half of the top 12 most highly expressed genes were located in the contigs mc66.3 (3 genes) or mc28.4 (3 genes); the rest were located in different contigs. Thus, scaffold 28 was the most variable and most highly expressed segment in the genome.

## Discussion

We used a comparative genomics approach to investigate the role of transposons in shaping the genome of the asexual fungal pathogen *F. virguliforme*. Transposons are known to play a major role in building two-speed genomes, with their variable compartments enriched in transposons [1–4]. The essential genome of *F. virguliforme* was drastically expanded through the generation of variable A+T-rich repeat blocks, as happened to the *L. maculans* genome [5, 6]. Most recently, the supernumerary genome of *F. virguliforme* was enriched in A+T-neutral transposons, one of which was active in a supernumerary segment whose SNP rate (between some *F. virguliforme* isolates) was 120 times higher than the average rate across the whole genome including the A+T-rich blocks. This complements the previous observation that the average non-synonymous substitution rate in the supernumerary chromosomes of *Mycosphaerella graminicola* was three times higher than in the essential chromosomes [8].

The scarceness of variation and the absence of A+T-neutral transposons in the essential genome contrast with the abundance of variation and the presence of A+T-neutral transposons in the supernumerary genome. This indicates that the activity of A+T-neutral transposons was inhibited much more strongly in the essential genome than in the supernumerary genome; the supernumerary genome provided places where A+T-neutral transposons could generate genetic variation much more rapidly without causing damage to the essential genome. Note that this rapid evolution occurred after the recent divergence of the *F. virguliforme* isolates; during that period, the A+T-rich repeat blocks in the essential genome, which were highly variable between closely related species, still had an extremely low SNP rate between the *F. virguliforme* isolates. Thus, the origin of the supernumerary genome was partly due to the origin of its transposons, which we addressed by looking at common sequences and transposons within and between *F. virguliforme* and its related species. Our results suggest that some transposons in the supernumerary genome moved between these species after the divergence of the *F. virguliforme* isolates. Moreover, an active DNA transposon was found in scaffold 28 with the most variable and highly expressed genes. This provides an explanation to the persistence of transposons as selfish DNA: transposons carry important genes (like ones encoding for P450 enzymes) and evolve them rapidly for their host without silencing them.

Supernumerary chromosomes are common in fungi, and they are hypothesized to originate by horizontal transfer [18]. Transposons are known to move by horizontal transfer [50]. Our results raised the possibility that this transfer is much more common than previously thought. If further studies confirm that horizontal transmission is common, this could have a dramatic and positive effect on our understanding of eukaryotic evolution.

The supernumerary segment in scaffold 28 of the *Fv* Mont-1 assembly was the most highly expressed as well as the most variable. This variation-expression connection suggests that variation is important to the success of this pathogen. Future studies on the functions of the genes in scaffold 28 might be able to shed light on the control and management of this pathogen.

## Supporting Information

S1 Fig Depths of coverage for contig mc28.2 by Illumina reads from each of the ten isolates. The figure consists of ten horizontal panels, one for each of the ten isolates in the same order as in Table 1. The top section in the panel shows coverage depths (peaks and valleys) as well as SNPs (color bars), with the range of coverage depths in a pair of square brackets at the upper left corner.
(PNG)

S2 Fig Depths of coverage for contig mc28.4 by Illumina reads from each of the ten isolates. (PNG)

S3 Fig Depths of coverage for contig mc74.1 by Illumina reads from each of the ten isolates. The contig region containing a gene encoding a cytochrome P450 enzyme was present in one *F. tucumaniae* isolate but not in the other *F. tucumaniae* isolates.
(PNG)
