# Supplementary figures and images for: Identification of Highly Variable Supernumerary Chromosome Segments in an Asexual Pathogen
Source: PLoS One. 2016 Jun 24;11(6):e0158183. doi: 10.1371/journal.pone.0158183 (PMC4920403; doi:10.1371/journal.pone.0158183)

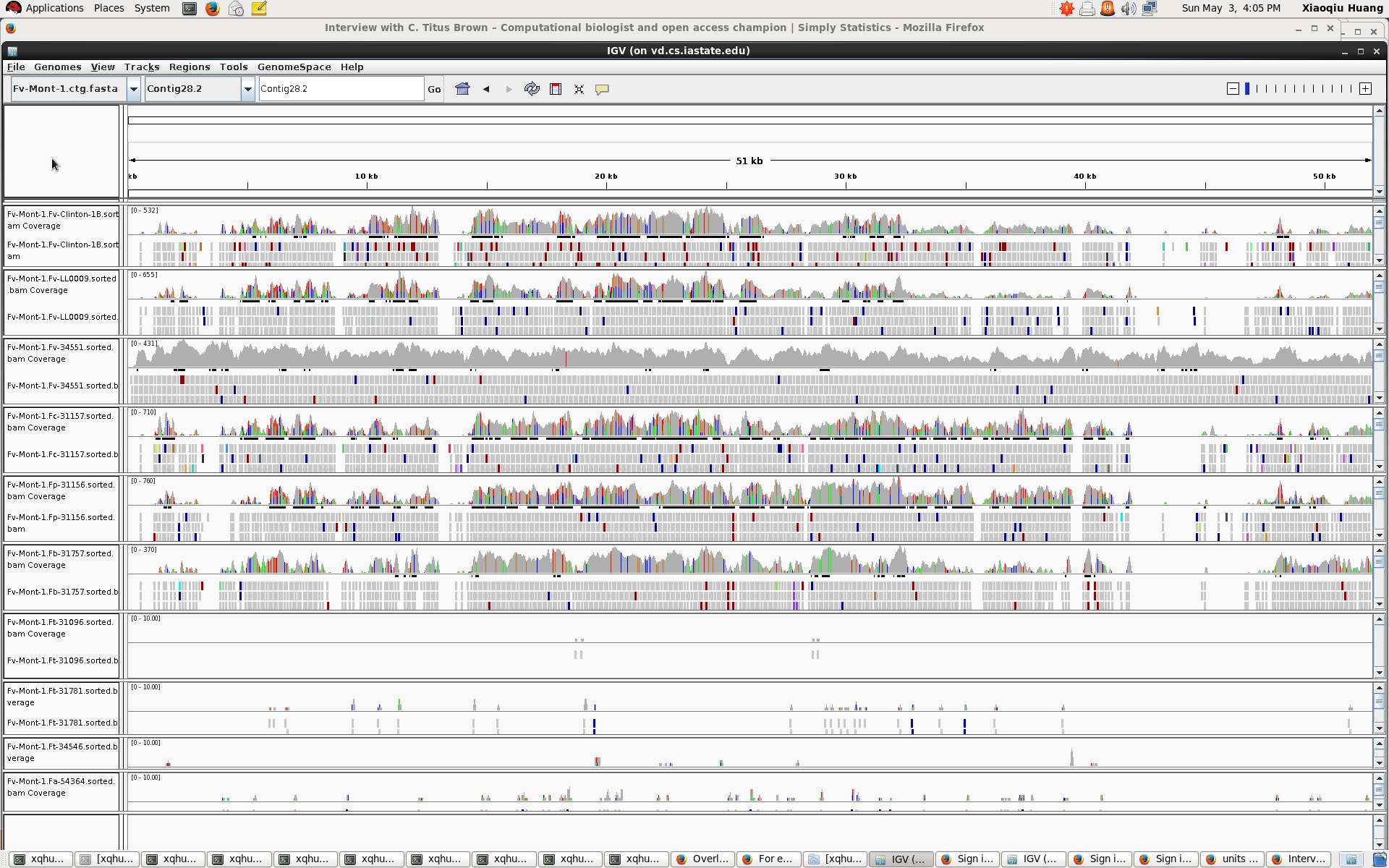

Supplement: S1 Fig — The figure consists of ten horizontal panels, one for each of the ten isolates in the same order as in Table 1. The top section in the panel shows coverage depths (peaks and valleys) as well as SNPs (color bars), with the range of coverage depths in a pair of square brackets at the upper left corner. (PNG) [file pone.0158183.s001.png]

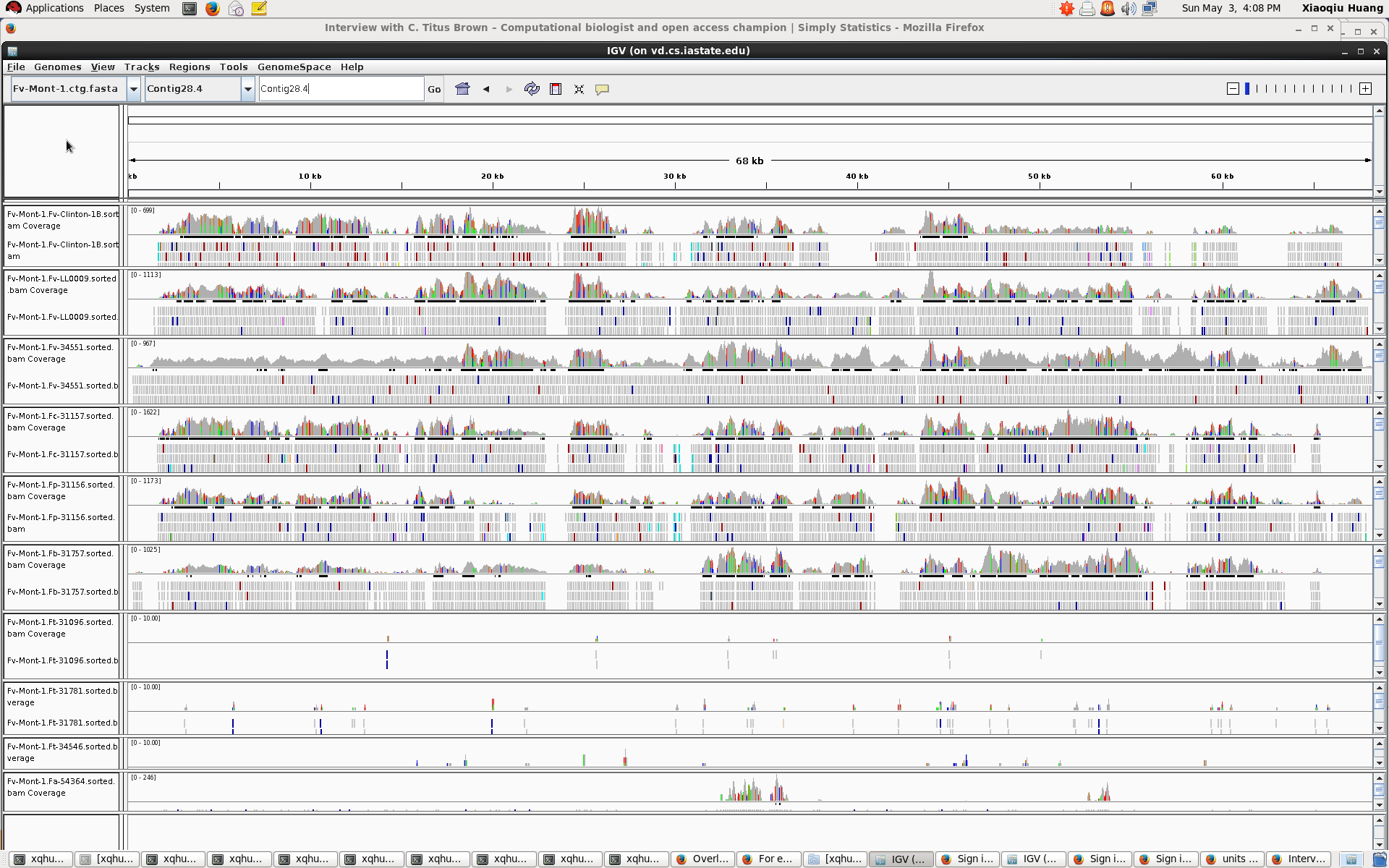

Supplement: S2 Fig — (PNG) [file pone.0158183.s002.png]

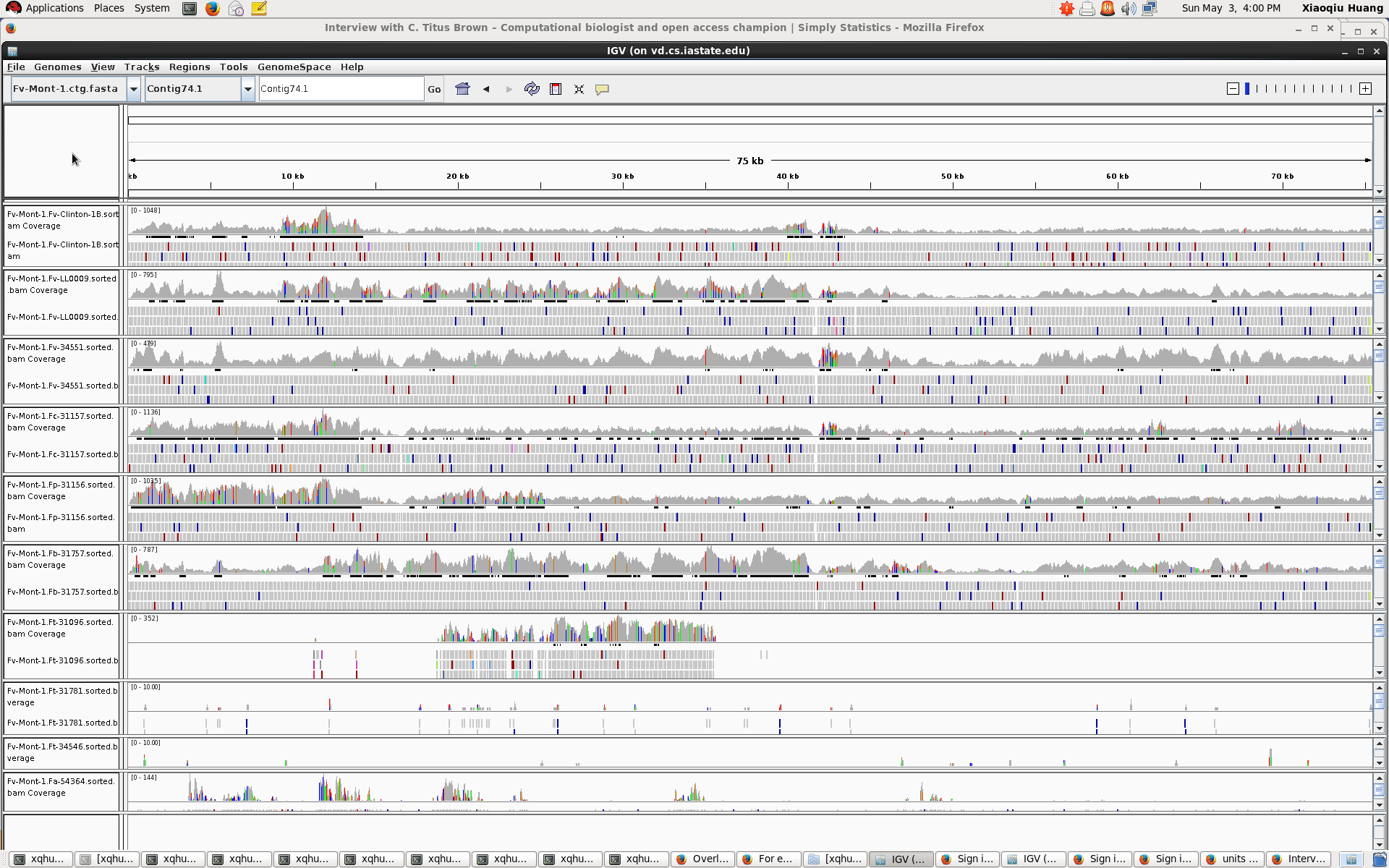

Supplement: S3 Fig — The contig region containing a gene encoding a cytochrome P450 enzyme was present in one F. tucumaniae isolate but not in the other F. tucumaniae isolates. (PNG) [file pone.0158183.s003.png]
